# Wear Assessment of Tibial Inserts Made of Highly Cross-Linked Polyethylene Supplemented with Dodecyl Gallate in the Total Knee Arthroplasty

**DOI:** 10.3390/polym13111847

**Published:** 2021-06-02

**Authors:** Min Zhang, Jia-Yu Wang, Jian Su, Jian-Jun Wang, Shi-Tong Yan, Yi-Chao Luan, Cheng-Kung Cheng

**Affiliations:** 1 Beijing Advanced Innovation Centre for Biomedical Engineering, School of Biological Science and Medical Engineering, Beihang University, Beijing 100083, China; m.zhang@buaa.edu.cn (M.Z.); wangjiayu626@163.com (J.-Y.W.); sujian@bimt.org.cn (J.S.); qluanyichao@163.com (Y.-C.L.); 2 Beijing Institute of Medical Device Testing, Beijing 101111, China; wangjianjun@bimt.org.cn (J.-J.W.); yanshitong@bimt.org.cn (S.-T.Y.); 3 School of Biomedical Engineering, Shanghai Jiao Tong University, Shanghai 200030, China

**Keywords:** wear particle, dodecyl gallate, highly cross-linked polyethylene, bone resorption, wear rate, wear test, total knee arthroplasty, implant fixation

## Abstract

Background: the wear of tibial insert is still one of primary factors leading to failure of total knee arthroplasty (TKA). Dodecyl gallate (DG) has shown improvements in the oxidation stability of highly cross-linked polyethylene (HXLPE). This study aimed to assess the application of HXLPE supplemented with DG (HXLPE-DG) on the tibial insert in TKA concerning the wear resistance and the potential impact on implant fixation; Methods: tibial inserts made of HXLPE-DG were subjected to a 3 million loading-cycle wear test following ISO 14243-1:2009. The loss of mass and wear rate of the tibial inserts were calculated. The quantity, size,- and shape of wear particles were recorded; Results: the test specimens lost an average mass of 16.00 mg ± 0.94 mg, and were on an average wear rate of 3.92 mg/million cycles ± 0.19 mg/million cycles. The content of wear particles in the calf serum medium was 3.94 × 10^8^ particles/mL ± 3.93 × 10^7^ particles/mL, 96.66% ± 0.77% of the particles had an equivalent circular diameter less than 0.5 μm. The aspect ratio of wear particles was 1.40 (min: 1.01; max: 6.42). Conclusions: HXLPE-DG displayed advantages over the commonly used materials for tibial inserts and presented the potential of application in TKA.

## 1. Introduction

Total knee arthroplasty (TKA) is considered to be an effective surgical treatment for knee osteoarthritis that can result in substantial improvements in knee function and patient quality of life [1]. However, the polymeric tibial insert is prone to wear due to the considerable loads placed upon it, which can lead to the generation of wear particles and subsequent loosening of the implant due to bone resorption [2,3]. Many methods have been used to improve the wear resistance in the previous reports [4,5,6]. Conventional ultra-high molecular weight polyethylene (UHMWPE), which was commonly used in clinic [7], was gradually replaced by highly cross-linked polyethylene (HXLPE) as the material of choice owing to its superior wear resistance [8]. Controlling the radiation atmosphere, the dose, and the dose rate could produce enough free radicals to highly cross-link polymer and consequently enhance wear resistance [9]. The cross-links could be formed by the recombination of the radicals created during the irradiation [10], and heat treatment after irradiation [4]. The downside was that cross-linking process lowered the oxidative resistance of the material, which was then combatted by supplementing the UHMWPE with antioxidant [4,5,11,12,13]. The addition of carbon nanofiller and paraffin oil in UHMWPE using ball milling and extrusion techniques has been reported to improve the wear resistance [5]. Vitamin E (or a-tocopherol) has been demonstrated effective in protecting irradiated UHMWPE against oxidation even at trace amounts [6,11,12,13,14,15,16]. However, long-term clinical data shows that the wear of the tibial insert is still one of the primary causes of revision surgery [17], due to oxidative damage to the polymer [18]. Thus, more efficient stabilizers may be demanded [6].

Tea polyphenols with multiple phenolic hydroxyls can scavenge more radiation-induced free radicals than vitamin E, which contains only one phenolic hydroxyl, and thus improve the oxidation stability of HXLPE [19]. Dodecyl gallate, a tea polyphenol containing three phenolic hydroxyls, has been shown to offer better protection to UHMWPE against oxidative damage than vitamin E [6]. Additionally, the HXLPE supplemented with dodecyl gallate (HXLPE-DG) has a comparable crystallinity, mechanical performance, and in-vitro biocompatibility to the UHMWPE with vitamin E [6]. Thus, the potential of this new material in the application to the tibial insert in TKA is interesting, regarding to the wear resistance and the effects of the wear particles on the implant fixation.

Wear particles generated from the gradual wearing of the tibial insert commonly lead to chronic inflammation, periprosthetic osteolysis, and eventually implant loosening or bone fracture [17]. The extent of the inflammatory biological reaction may be linked to the quantity, size, and morphology of the wear debris [20]. Rough wear debris with a sharp and elongated shape may provoke particularly acute periprosthetic inflammation [20]. 

This study aimed to assess the wear properties of tibial inserts made of HXLPE-DG and to evaluate how the generated wear debris may affect implant fixation. A total knee replacement with an HXLPE-DG tibial insert was loaded with a knee simulator and the subsequent wear loss, wear rate, and characteristics of wear particles (quantity, size, and shape) were used to assess implant stability. It was hypothesized that the HXLPE-DG tibial insert would demonstrate superior wear resistance to other materials commonly used for the tibial insert (i.e., UHMWPE, HXLPE, highly cross-linked polyethylene supplemented with vitamin E (HXLPE-VE)) [8,17] and that the characteristics of the wear debris would put the surrounding bone at low risk of particle-induced periprosthetic inflammation, osteolysis, and implant loosening.

## 2. Materials and Methods

### 2.1. Wear Test

#### 2.1.1. Prosthesis

**Figure 1:**
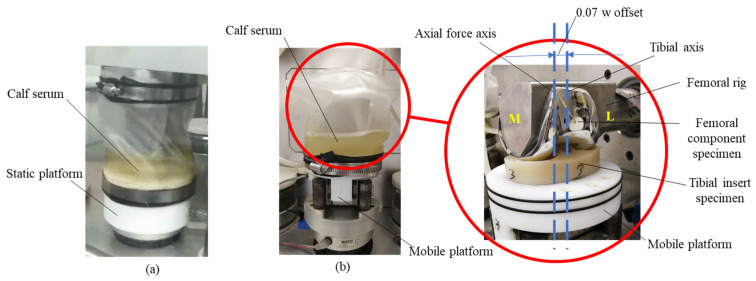
Setup of the wear test with the AMTI knee simulator. (**a**) Setup of the control specimen in the knee simulator. The control specimen was immersed in calf serum (20 g/L) throughout the wear test and subject to the same time-varying axial force as the test specimen, intending to determine the amount of mass change due to fluid transfer. (**b**) Setup of the test specimen in the knee simulator. The test specimen was immersed in calf serum (20 g/L) throughout the wear test and subject to a loading condition including axial force, anterior-posterior (AP) force, rotation torque, and flexion angle, in order to test the implant wear (M: Medial; L: Lateral).

The knee replacement used in this study was a posterior-stabilized (PS) system design (Diamond TKA implant, Beijing Naton Technology Group Company Ltd., Beijing, China). The femoral component was made of CoCrMo and all the testing tibial bearing inserts were made of HXLPE-DG with a size 83.1 mm × 54.1 mm × 18.0 mm (working accuracy: 0.5 mm; roughness: 0.1 µm) (Diamond TKA implant, Beijing Naton Technology Group Company Ltd., Beijing, China). The method for producing the material of HXLPE-DG reported by Fu et al. [6] was followed in this study. The dodecyl gallate (Nanjing Duly Biotech Co., Ltd., Nanjing, China) was blended with medical-grade UHMWPE powders (GUR 1050, Orthoplastics, Lancashire, UK) at 0.05 wt% (weight percent) and consolidated at 190 °C. The consolidated blocks were vacuum-packaged and irradiated with a 10 MeV electron beam (Huaneng Electron Accelerator Company, Shaoxing, China) at 25 kGy per pass at room temperature to a total dose of 100 kGy [6]. With reference to ISO 14243-1:2009 [21], which was a standard guideline for wear test of total knee-joint prosthesis, four implants were used for the test, three of which were randomly selected as test specimens, and the remaining was a control specimen for the calculation of wear loss and wear rate (Figure 1).

#### 2.1.2. Test Condition

The wear test was carried out on an AMTI knee simulator (ADL-K6-01, Advanced Mechanical Technology Inc, Watertown, MA, USA) (accuracy: 5%) (Figure 1). The femoral component was secured on a femoral rig and connected to an actuator on the simulator which could rotate around its axis and simulate the knee flexion. The tibial insert was mounted to a mobile platform with a tibial rig which could simulate knee rotation around the tibial axis as well as apply axial and anterior-posterior (AP) forces (Figure 1b). Following ISO 14243-1:2009 [21], the tibial insert was placed slightly lateral to the axis of the mobile platform at a distance of 0.07 times the width of the tibial component (5.8 mm in the test) (Figure 1b). All three test specimens were subjected to an axial force, anterior-posterior (AP) force, rotation torque, and flexion angle using input values recommended in ISO 14243-1:2009 [21] (Supplementary Materials). The axial force applied to the tibial component was positive when it acted from inferior to superior. The AP force was positive when it acted from posterior to anterior. The rotation torque was positive when the tibial rotated internally. The implants used in this study were all left-sided, thus the rotation torque was positive when it acted clockwise. The flexion angle is considered the relative angular motion between the femoral and tibial components, with an increase in the flexion angle being positive. Using the knee simulator machine, the axial force, rotation torque, and AP force were applied to the tibial component and the flexion force was applied to the femoral component. The tibia component was constrained in flexion-extension, while the femoral component was only free to move in flexion-extension during the gait simulation. 

The wear test was conducted following ISO 14243-1:2009 [21]. The knee simulator was operated at a frequency of 1 Hz. The axial force applied by the machine was maintained within a tolerance of 1° parallel to the tibial axis. The axial force, flexion/extension motion, AP force, and tibial rotation torque were all maintained within a tolerance of ±5% from the maximum value and ±3% of the full cycle time specified throughout the cycle. Both the test specimens and control specimens were immersed in calf serum (20 g/L) (Thermo Fisher Scientific corporation, Waltham, MA, USA) throughout the wear test. The serum was changed every 500,000 cycles. The temperature of the serum was controlled at 37 °C using an antiseptic agent consisting of 0.2% NaN_3_ and 5 mmol/L Ethylenediaminetetraacetic acids (EDTA). The test was stopped for measurements after 500,000 cycles, 1 million cycles, 2 million cycles, and 3 million cycles. The test was ceased after 3 million cycles [22].

At each testing stage (500,000, 1 million, 2 million, and 3 million cycles), the three tibial insert test specimens and one control specimen underwent gravimetric measurement following ISO 14243-2:2016 [23]. An analytical balance (Mettler Toledo AX205DR, Mettler-Toledo International Inc, Greifensee, Switzerland) (accuracy: 0.01 mg) was used to measure the mass of each specimen three times, and the average value was considered the mass of the specimen. 

### 2.2. Wear Assessment

#### 2.2.1. Gravimetric Wear and Wear Rate

The gravimetric wear and wear rate were calculated according to ISO 14243-2:2016 [23]. The gravimetric wear ($W_{n}$) referred to the net loss of mass from each test specimen after n cycles of loading and was calculated using Equation (1). $m_{0}$ was the mass of the test specimen before the wear test. $m_{n}$ was the mass of the test specimen after n loading cycles. $S_{n}$ was the mass change due to the fluid, and obtained from the increase in mass of the control specimen over the same period. $S_{n}$ was related to $\bar{m_{0}}$ and $\bar{m_{n}}$. $\bar{m_{0}}$ was the mass of the control specimen before the wear test and $\bar{m_{n}}$ was the mass of the control specimen after n loading cycles. The gravimetric wear of all the test specimens was calculated at each testing stage during the wear simulation (500,000, 1 million, 2 million, and 3 million cycles).

(1) $$\begin{array}{c} W_{n}=m_{0}-m_{n}+S_{n}\\ S_{n}=\bar{m_{0}}-\bar{m_{n}} \end{array}$$

The wear rate referred to the least-squares linear fit relationship between $W_{n}$ and the number of loading cycles (n). The wear rate of each specimen after the 3 million loading cycles was calculated. The values of the three test specimens were averaged and represented the wear rate of the HXLPE-DG tibial insert.

#### 2.2.2. Wear Particle Analysis

Wear particles were collected after 1 million, 2 million, and 3 million cycles during the wear simulation and evaluated regarding the quantity, size, and morphology. The collection and assessment processes were detailed below. 

The particles were firstly isolated by methods specified in ISO 17853:2011 [24], which was a standard guideline for isolation and characterization of polymer wear particles. At each testing stage, 10 mL of the calf serum medium for each test implant was respectively mixed with 40 mL of hydrochloric acid (volume fraction: 37%) and stirred for one hour at a temperature of 50 °C to dissolve any proteins. 0.5 mL of each prepared solution was filtered using microporous organic filter membrane (polycarbonate) (Sterlitech Corporation, Kent, WA, USA) with an aperture of 1 µm to collect particles larger than 1 µm. The remaining solution was then filtered using 0.1 µm filter membrane to collect particles between 0.1 µm and 1 µm, in which range the particles have been implicated in the onset of bone osteolysis [25]. 

Secondly, the collected particles were characterized by ISO 17853:2011 [24]. The filter membrane containing filtered particles was dried and scanned using a scanning electron microscope (SEM) (JEOL JSM-6700F, JEOL Ltd. Tokyo, Japan) (Resolution: 1.0 nm) with an accelerating voltage of 10 keV. Twenty non-overlapping fields of view on the filter membrane with the 0.1 µm–1 µm wear particles were randomly captured [26]. The images were captured at a magnification of 10,000 times [24]. The HXLPE-DG particles in all the twenty images were identified in an image analysis software (ImageJ, contributors worldwide, USA). 

The quantity of the HXLPE-DG particles was recorded using ImageJ (Contributors worldwide, USA), and used to calculate the total number of HXLPE-DG particles per milliliter of the calf serum medium from the test specimens. Firstly, the number of wear particles presented on the filter membrane was calculated by using the method reported by Markhoff et al. [8] (Equation (2)). In Equation (2), N is the quantity of HXLPE-DG particles on the filter membrane; n is the quantity of HXLPE-DG particles from all twenty SEM images; *S* is the area of the filter membrane; *A* is the area of each SEM image. As the wear particles on each filter membrane were from 0.1 mL of the calf serum medium from the test specimens in this study, the content of wear particles in the calf serum medium was then calculated and recorded at each test stage (1 million, 2 million, and 3 million cycles).

(2) $$N=n\times \frac{S}{20\times A}$$

The size of the HXLPE-DG wear particles was determined using the equivalent circular diameter (ECD) method detailed in ASTM F1877-05 [27]. The ECD refers to the diameter of a circle with the same area as the particle. The ECD of each particle on each SEM image was calculated at each test stage (1 million, 2 million, and 3 million cycles). 

The aspect ratio (AR) was the ratio of the maximum to the minimum diameter of the projection of each particle and was used to assess the morphology of the particles [27]. The AR of each particle on each SEM image at each test stage was calculated using ImageJ software (ImageJ, contributors worldwide, USA). Considering that sharp and elongated particles (2.4 ≤ AR < 5) may induce adverse cellular activities and provoke periprosthetic inflammation [20], the percentage of particles with AR ≥ 2.4 was calculated at each test stage (1 million, 2 million, and 3 million cycles).

Shapiro-Wilk test was used to perform the normality test, and data was presented as a mean (standard deviation (SD)) for normally distributed continuous variables, and a median (minimum, maximum) for non-normally distributed continuous variables. A priori power analysis with a significance level of 0.05 (type-I error), the desired power of 80%, and the effect size of 0.5 indicating a large difference was performed to evaluate the sample size. The variance in three test stages was analyzed by using Kruskal-Wallis one-way analysis of variance for the nonparametric variables. Differences were considered significant at *p* values < 0.05.

## 3. Results

### 3.1. Gravimetric Wear and Wear Rate

The average gravimetric wear of the test samples during the wear test is given in Table 1. It showed that the wear increased with the number of cycles. The average gravimetric wear across the three test specimens was 16.00 mg ± 0.94 mg after three million cycles. The average wear rate was 3.92 mg/million ± 0.19 mg/million cycles with a correlation coefficient of 0.96 ± 0.03.

### 3.2. Measurement of Wear Particles

**Figure 2:**
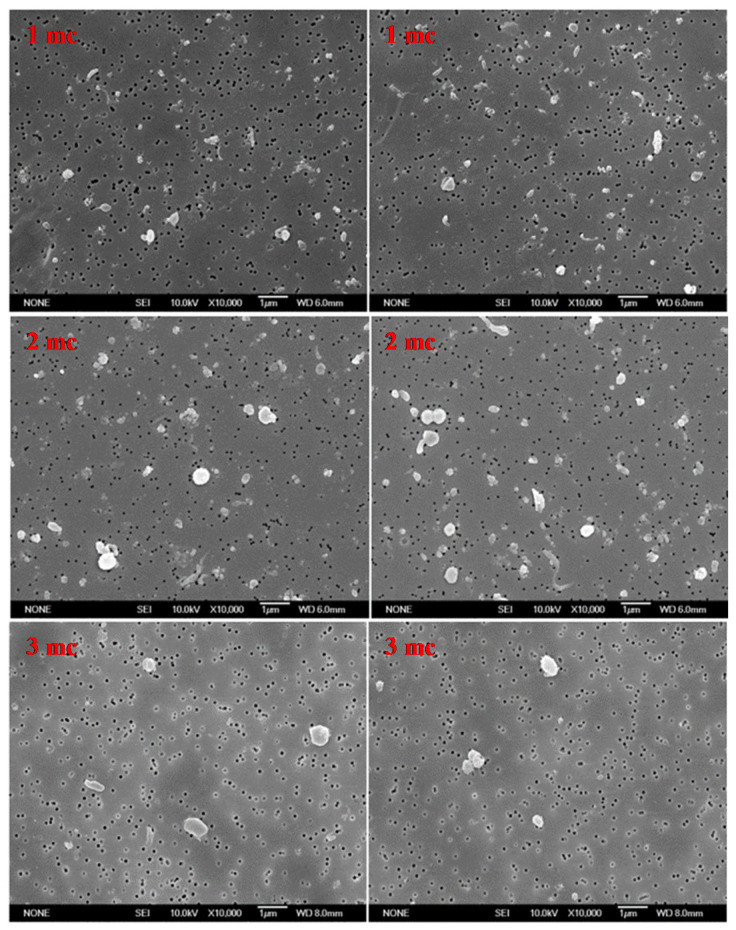
Two SEM images captured after 1million, 2 million and 3 million cycles. mc: million cycles.

Two SEM images after every one million cycles were randomly collected to be displayed in Figure 2. It was found that the polyethylene particles generally became bigger and more spherical with the number of wear cycles.

**Figure 3:**
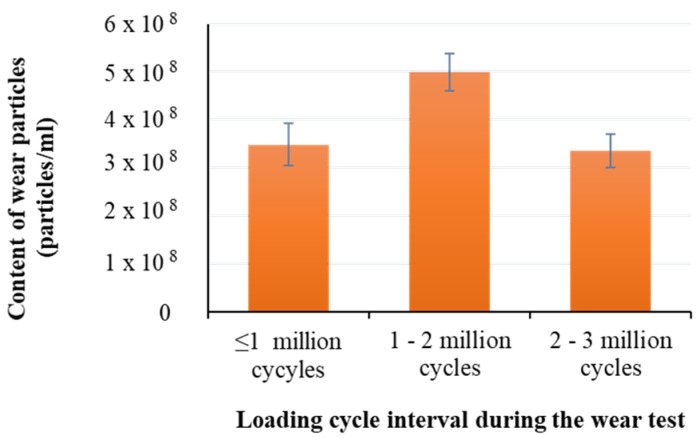
Content of wear particles generated after every one million cycles.

The quantity of HXLPE-DG particles presented on the filter membrane after every million cycles are shown in Figure 3. The results showed that the number of wear particles increased when going from one million cycles ($3.48\times 10^{8}\mathrm{particles}/\mathrm{mL}\pm 4.36\times 10^{7}\mathrm{particles}/\mathrm{mL}$) to two million cycles ($4.99\times 10^{8}\;\mathrm{particles}/\mathrm{mL}\pm 3.89\times 10^{7}\;\mathrm{particles}/\mathrm{mL})$, but then reduced to $3.35\times 10^{8}\;\mathrm{particles}/\mathrm{mL}\pm 3.53\times 10^{7}\mathrm{particles}/\mathrm{mL}\;$when measured at three million cycles (*p* < 0.001). The total number of wear particles across the three million cycles was $1.18\times 10^{8}\pm 1.18\times 10^{7}$, with the content of wear particles in the calf serum medium being $3.94\times 10^{8}\;\mathrm{particles}/\mathrm{mL}\pm 3.93\times 10^{7}\;\mathrm{particles}/\mathrm{mL}.$

**Figure 4:**
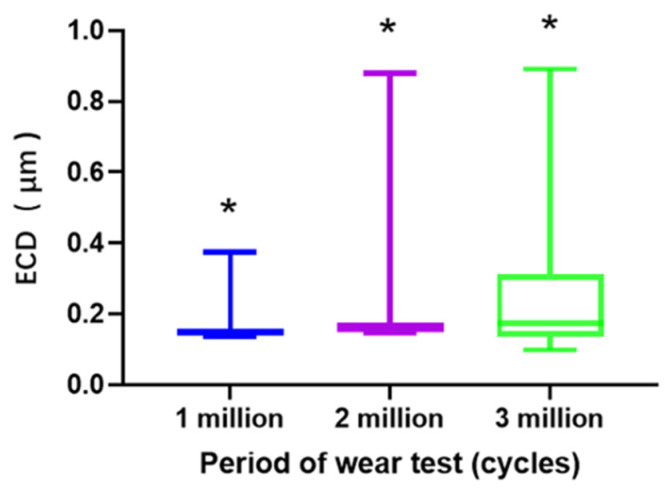
Size of wear particles measured by equivalent circular diameter (ECD), (*: *p* < 0.05).

**Figure 5:**
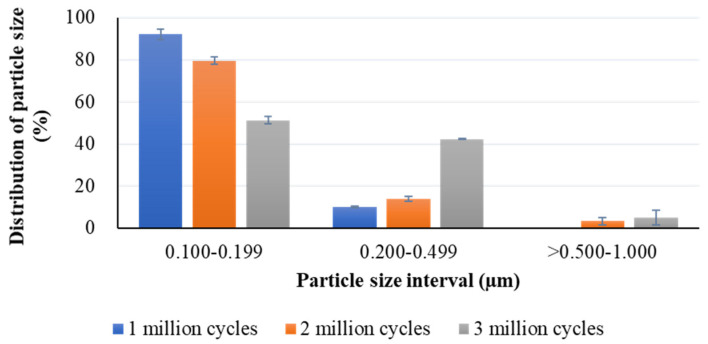
Distribution of wear particle size for each size interval.

The size of the wear particles was determined using the equivalent circular diameter (ECD). The results of Shapiro-Wilk test showed that the data of ECD was non-normally distributed. There was a significant difference in the size of the wear particles at the three test stages (one million, two million, and three million loading cycles) (*p* < 0.001) (Figure 4). Moreover, the wear particles became large in size as the load cycles increased, giving a median ECD of 0.15 μm (min: 0.14 μm; max: 0.38 μm) at one million cycles, 0.16 μm (min: 0.15 μm; max: 0.88 μm) at two million cycles, and 0.17 μm (min: 0.01 μm; max: 0.89 μm) at three million cycles (Figure 4). When summarized the size of wear particles into three intervals (0.10 μm–0.19 μm; 0.20 μm–0.49 μm; > 0.50 μm), it found that the particles within 0.20 μm in size covered 92.04% ± 2.60% of research particles at one million cycles, 79.58% ± 1.87% at two million cycles, and 51.32% ± 1.86% at three million cycles (Figure 5). Additionally, the distribution of the particles with the ECD less than 0.5 μm after three million cycles reached 96.66% ± 0.77%. 

**Figure 6:**
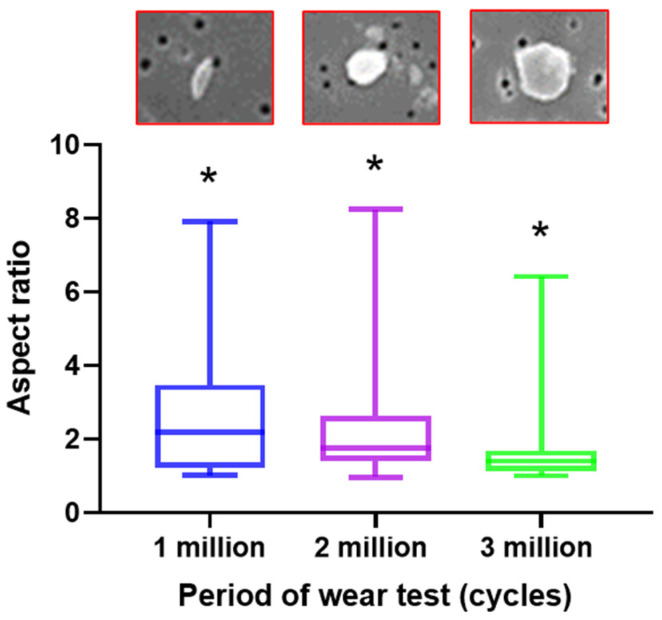
Distribution of aspect ratio after every one million cycles, (*: *p* < 0.05).

The shape of the HXLPE-DG wear particle was evaluated with an aspect ratio (AR). It was found that the data of AR was also non-normally distributed. The boxplot in Figure 6 showed a significant difference in the distribution of AR in the test stages (one million, two million, and three million loading cycles) (*p* = 0.005). The median value of AR was 2.19 (min: 1.01; max: 7.92) at one million loading cycles, 1.77 (min: 0.97; max: 8.32) at two million loading cycles, and 1.40 (min: 1.01; max: 6.42) at three million loading cycles, signifying a change in the shape of the wear particles from an elongated to spherical shape. The percentage of particles with AR ≥ 2.4 (presenting sharp and elongated shape) decreased as the test progressed, being 44.01% ± 1.76% at one million cycles, 28.07% ± 2.45% at two million cycles, and 13.2% ± 1.63% at three million cycles.

## 4. Discussion

The material of tea polyphenols has shown improvement in the oxidative resistance of highly cross-linked UHMWPE [19]. As such, this study aimed to investigate the wear properties of HXLPE blended with dodecyl gallate, a tea polyphenol containing three phenolic hydroxyls, and evaluate the application of this material on the knee insert in TKA. The key findings of this study were: (1) After three million loading cycles, the gravimetric wear of the HXLPE-DG tibial insert was 16.00 mg ± 0.94 mg and the wear rate was 3.92 mg/million cycles ± 0.19 mg/million cycles, indicating advantages in wear resistance over traditional UHMWPE (the gravimetric wear: 62.00 mg [28]; wear rate: 19.88 mg/million cycles [28]); (2) The size of the wear particles generally increased with the number of loading cycles and 96.66% ± 0.77% of the particles had an ECD of less than 0.5 μm; (3) The aspect ratio (AR) of the particles with sizes ranging from 0.1 μm to 1 μm decreased with the increasing number of wear cycles, and the shape of the particles changed from an elongated shape to a spherical shape.

In this study, three tibial inserts made of HXLPE-DG were tested to assess their wear resistance. The wear rate in our study (3.92 mg/million cycles ± 0.19 mg/million cycles) was higher than that reported by Fu et al. (2.29 mg/million cycles ± 0.31 mg/million cycles) [6], although the same material was tested. This difference may be explained by the design of the specimens and the loading conditions used in the wear test. In our study, the tibial inserts replicated the design of a commercially available prosthesis (Diamond TKA implant, size 5, Beijing Naton Technology Group Company Ltd., Beijing, China), while Fu et al. used pin-shaped samples. Additionally, the loading conditions in our study simulated natural gait following ISO 14243-1:2009 [21], which was a more complex loading system than that used by Fu et al. [6]. In comparison to other materials, the wear rate of the HXLPE-DG in this study was higher than that for UHMWPE (wear rate: approximately 19.88 mg/million cycles ± 4.00 mg/million cycles) [28], and comparable to the rate reported for the HXLPE blended with vitamin E (HXLPE-VE) (approximately 2.70 mg/million ± 1.60 mg/million) with the same testing method and the same type of knee insert design (Posterior Stabilized (PS) design) [28]. The addition of dodecyl gallate to the insert material improved the wear resistance over UHMWPE and reflect comparable advantages on the wear resistance over vitamin E within three million cycles of wear test. 

The number of wear particles deposited in the serum medium after every million cycles during the wear test was assessed. It was found that the number of wear particles increased between one million and two million cycles, but then decreased when loaded to three million cycles. The increased quantity of the wear particles within the two million cycles may be due to the initial relative rough contact surfaces between the tibial and femoral components, and when the contact between the two components reached to steady state, the quantity of the wear particles reduced. In our study, the average number of wear particles released across the three million cycles was 1.18 × 10^8^ ± 1.18 × 10^7^. If it assumed that the wear rate after three million cycles was similar to the wear rate after five million cycles [28], the predicted number of wear particles after five million cycles would be 2.30 × 10^8^. This value was lower than the quantity reported for HXLPE-VE (5.5 × 10^8^), UHMWPE (2.6 × 10^9^), and HXLPE (1.7 × 10^9^) in the same amount of calf serum medium across five million cycles of wear test [8]. 

Figure 5 shows that the particles within 0.20 μm in size covered 92.04% ± 2.60% of research particles at one million cycles, 79.58% ± 1.87% at two million cycles, and 51.32% ± 1.86% at three million cycles. Additionally, 96.66% ± 0.77% of wear particles had an ECD of less than 0.5 μm. This finding indicated that the size of wear debris from HXLPE- DG was comparable with those from the materials commonly used for tibial insert in TKA (i.e., UHMWPE, HXLPE, and HXLPE-VE) [8]. Markhoff et al. [8] reported that the ECD of most wear particles from UHMWPE, HXLPE, and HXLPE-VE were between 0.1 μm and 0.2 μm. Moreover, Markhoff et al. [8] also found that the ECD of wear particles from UHMWPE, HXLPE, and HXLPE-VE increased from one million to three million loading cycles, which was consistent with the findings from HXLPE- DG in this current study. The particles observed in the SEM images after three million cycles were noticeably larger than those after one million cycles. 

The aspect ratio (AR) was used to identify the shape of wear particles using the methods detailed in ASTM F 1877-05 [27]. The results showed that the AR of the particles reduced as the number of loading cycles increased. The roundness of particles is inversely proportional to the AR value, and so it could be concluded that the particles became more spherical as the test progressed. This can be also observed in the SEM images in Figure 2. The increasing roundness of the particles could be a result of the combined loading placed on the knee, particularly the rotation torque. The mean AR of all the particles in this study was 1.40 (min: 1.01; max: 6.42) after three million cycles and was predicted to be a smaller value after five million cycles, as this study indicated that AR reduced with the increased loading cycles. The AR observed in this study was smaller than the reports for UHMWPE (2.1), HXLPE (1.7), and HXLPE-VE (2.0) after five million cycles [8], displaying a rounder shape. It was reported that cross-linking may be associated with decreased deformability of polyethylene [14]. Furthermore, an increased radiation dose could be another reason for the reducing number of fibrillar wear particles [29]. The rounder wear particles produced by the material of HXLPE-DG displayed the potential of reducing acute adverse tissue responses [20]. The percentage of particles with the AR ≥ 2.4 (representing an elongated shape) decreased as the test progressed, being 44.01% ± 1.76% at one million cycles, 28.07% ± 2.45% at two million cycles, and 13.2% ± 1.63% at three million cycles. This may explain why bone osteolysis is often reported in the early follow-up stages after orthopedic implantation [30]. 

There are some limitations with this study that should be noted. Firstly, the number of samples for the wear testing was relatively small (only 4). Although the number of test samples satisfied the requirement of ISO 14243-1:2009, a larger number of samples may improve the accuracy of the calculations for wear mass loss and wear rate. Secondly, the wear test in this study was conducted up to three million cycles. A longer simulation may be considered in future studies to evaluate implant function at long-term follow-up. Thirdly, the wear test in this study was carried out by ISO 14243-1:2009 and the bovine serum containing wear particles was replaced every million cycles, but this method limits the evaluation of third-body wear. It has been reported that third-bodies may increase the generation of particles and slightly alter the particle morphology, resulting in a heightened inflammatory response [26]. A method that considers the third-body wear may improve the accuracy of the wear assessment and will be performed in future studies. Fourthly, an in vitro experiment using macrophages and wear particles will be performed to further investigate the extent of the inflammatory biological reaction induced by the wear particles from the knee insert made of the highly cross-linked polyethylene supplemented with dodecyl gellate in future. Fifthly, considering the reported advantages of HXLPE-DG on oxidation stability, the long-term wear-resistance of a tibial insert made of this material in the condition of aging to accelerate oxidative degradation would be further studied. Finally, only the wear resistance and effects of the wear particles on the implant fixation were assessed in current study, the potential application of the tibial insert with HXLPE-DG regarding to other properties (i.e., infection, comorbidity) will be further investigated in future. 

## 5. Conclusions

The material of HXLPE-DG was superior to UHMWPE in wear resistance, the quantity of wear debris, and wear particle shape, putting the surrounding bone at low risk of particle-induced osteolysis. Furthermore, HXLPE-DG produced fewer wear particles and displayed a rounder shape, thus could reduce acute adverse tissue responses by comparison with HXLPE and HXLPE-VE, and subsequently improving the implant stability, thus presented potential application on the tibial inserts in TKA. 

## Figures and Tables

**Table 1:** Gravimetric wear and wear rate.

Number of Cycles	Gravimetric Wear (mg)	Wear Rate (mg/Million Cycles)	Correlation Coefficient (R^2^)
0.5 million	3.30 ± 0.51	-	-
1 million	6.24 ± 2.51	-	-
2 million	10.40 ± 1.50	-	-
3 million	16.00 ± 0.94	3.92 ± 0.19	0.96 ± 0.03

## Data Availability

The data is available from the corresponding author.

## Supplementary Materials

The following are available online at https://www.mdpi.com/article/10.3390/polym13111847/s1, Figure S1: Loading in one cycle of gait movement. (a) Variation of flexion angle with time. (b) Variation of axial force with time. (c) Variation of anteroposterior (AP) force with time. (d) Variation of rotation torque with time.

## Abbreviations

TKA	total knee arthroplasty
DG	dodecyl gallate
HXLPE	highly cross-linked polyethylene
HXLPE-DG	highly cross-linked polyethylene supplemented with dodecyl gallate
UHMWPE	Conventional ultra-high molecular weight polyethylene
HXLPE-VE	highly cross-linked polyethylene supplemented with vitamin E
