# Supplementary material for: Wear Assessment of Tibial Inserts Made of Highly Cross-Linked Polyethylene Supplemented with Dodecyl Gallate in the Total Knee Arthroplasty
Source: Polymers (Basel). 2021 Jun 2;13(11):1847. doi: 10.3390/polym13111847 (PMC8199669; doi:10.3390/polym13111847)
Supplement: Supplementary file 1 [file polymers-13-01847-s001.zip › polymers-1211156-supplementary.pdf]

polymers

MDPI

Supplementary Material: Wear Assessment of Tibial Inserts Made of Highly Cross-Linked Polyethylene Supplemented with Dodecyl Gallate in the Total Knee Arthroplasty

Min Zhang, Jia-Yu Wang, Jian Su, Jian-Jun Wang, Shi-Tong Yan, Yi-Chao Luan and Cheng-Kung Cheng

Loading in one cycle of gait movement

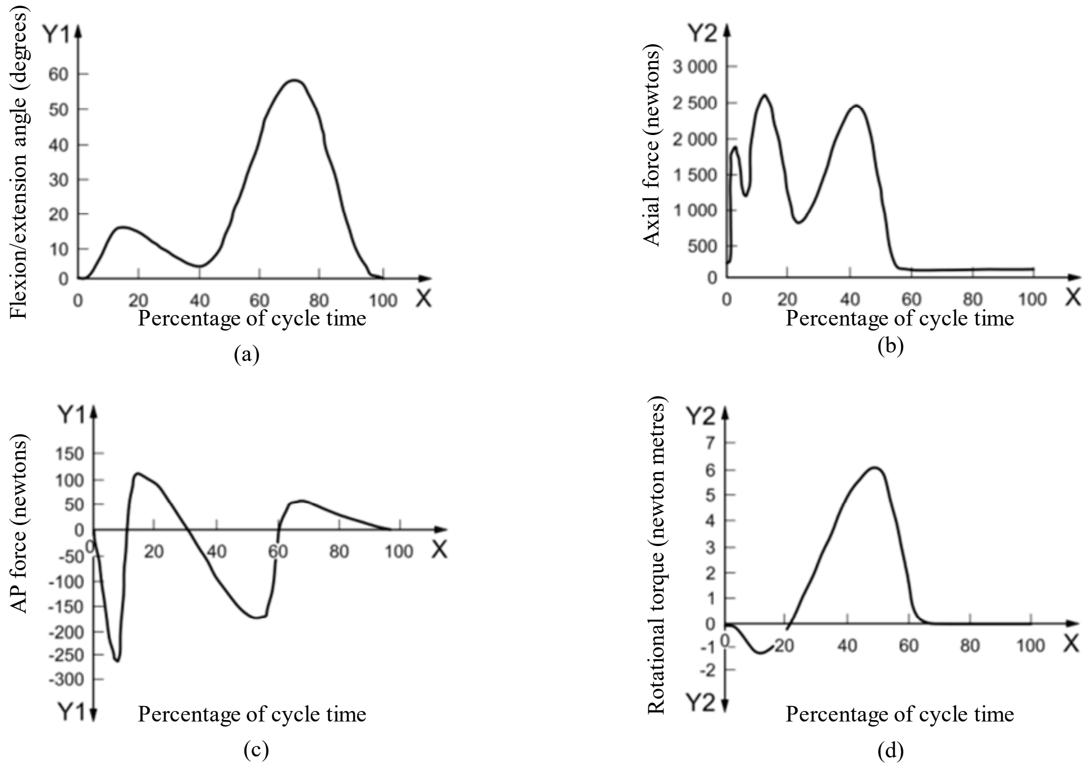

Figure S1. Loading in one cycle of gait movement [1]. (**a**) Variation of flexion angle with time. (**b**) Variation of axial force with time. (**c**) Variation of anteroposterior (AP) force with time. (**d**) Variation of rotation torque with time.

Reference

1. ISO 14243-1, Implants for Surgery—Wear of Total Knee-Joint Prostheses. In *Part 1: Loading and Displacement Parameters for Wear-Testing Machines with Load Control and Corresponding Environmental Conditions for Test;* ISO Copyright Office: Geneva, Switzerland, 2009.
